# Potential of marker selection to increase prediction accuracy of genomic selection in soybean (*Glycine max* L.)

**DOI:** 10.1007/s11032-016-0504-9

**Published:** 2016-07-28

**Authors:** Yansong Ma, Jochen C. Reif, Yong Jiang, Zixiang Wen, Dechun Wang, Zhangxiong Liu, Yong Guo, Shuhong Wei, Shuming Wang, Chunming Yang, Huicai Wang, Chunyan Yang, Weiguo Lu, Ran Xu, Rong Zhou, Ruizhen Wang, Zudong Sun, Huaizhu Chen, Wanhai Zhang, Jian Wu, Guohua Hu, Chunyan Liu, Xiaoyan Luan, Yashu Fu, Tai Guo, Tianfu Han, Mengchen Zhang, Bincheng Sun, Lei Zhang, Weiyuan Chen, Cunxiang Wu, Shi Sun, Baojun Yuan, Xinan Zhou, Dezhi Han, Hongrui Yan, Wenbin Li, Lijuan Qiu

**Affiliations:** 1 College of Agriculture, Northeast Agricultural University, Harbin, 150030 China; 2 The National Key Facility for Crop Gene Resources and Genetic Improvement (NFCRI), Institute of Crop Sciences, Chinese Academy of Agricultural Sciences, Beijing, 100081 China; 3 Soybean Research Institute, Heilongjiang Academy of Agricultural Sciences, Harbin, 150086 China; 4 Department of Breeding Research, Leibniz Institute of Plant Genetics and Crop Plant Research (IPK), 06466 Gatersleben, Germany; 5 Department of Plant, Soil and Microbial Sciences, Michigan State University, East Lansing, MI 48824 USA; 6 Institute of Crop Sciences, Chinese Academy of Agricultural Sciences, Beijing, 100081 China; 7 Heilongjiang Academy of Agricultural Sciences, Harbin, 150086 China; 8 Soybean Research Institute, Jilin Academy of Agricultural Sciences, Changchun, 130033 China; 9 Chifeng Institute of Agricultural Sciences, Chifeng, 024031 China; 10 Institution of Cereal and Oil Crops Hebei Academy of Agricultural and Forestry Sciences, Shijiazhuang, 050031 China; 11 Economic Crops Institute, Henan Academy of Agricultural Sciences, Zhengzhou, 450002 China; 12 Crop Research Institute, Shandong Academy of Agricultural Sciences, Jinan, 250010 China; 13 Oil Crop Research Institute, Chinese Academy of Agricultural Sciences, Wuhan, 430062 China; 14 Institute of Crop Sciences, Jiangxi Academy of Agricultural Sciences, Nanchang, 330200 China; 15 Institute of Economical Crops, Guangxi Academy of Agricultural Sciences, Nanning, 530007 China; 16 Hulun Buir Institute of Agricultural Sciences, Hulun Buir, 021000 China; 17 Heihe Branch Institute, Heilongjiang Academy of Agricultural Sciences, Heihe, 164300 China; 18 The Crop Research and Breeding Center of Land-Reclamation, Harbin, 150090 Heilongjiang China; 19 Suihua Branch Institute, Heilongjiang Academy of Agricultural Sciences, Suihua, 152052 China; 20 Jiamusi Branch Institute, Heilongjiang Academy of Agricultural Sciences, Jiamusi, 154007 China; 21 Crop Institute, Anhui Academy of Agricultural Sciences, Hefei, 230031 Anhui China; 22 Zhoukou Institute of Agricultural Sciences, Zhoukou, 466001 Henan China

**Keywords:** Genomic selection, Prediction accuracy, *Glycine max*, Sampling method

## Abstract

Genomic selection is a promising molecular breeding strategy enhancing genetic gain per unit time. The objectives of our study were to (1) explore the prediction accuracy of genomic selection for plant height and yield per plant in soybean [*Glycine max* (L.) Merr.], (2) discuss the relationship between prediction accuracy and numbers of markers, and (3) evaluate the effect of marker preselection based on different methods on the prediction accuracy. Our study is based on a population of 235 soybean varieties which were evaluated for plant height and yield per plant at multiple locations and genotyped by 5361 single nucleotide polymorphism markers. We applied ridge regression best linear unbiased prediction coupled with fivefold cross-validations and evaluated three strategies of marker preselection. For plant height, marker density and marker preselection procedure impacted prediction accuracy only marginally. In contrast, for grain yield, prediction accuracy based on markers selected with a haplotype block analyses-based approach increased by approximately 4 % compared with random or equidistant marker sampling. Thus, applying marker preselection based on haplotype blocks is an interesting option for a cost-efficient implementation of genomic selection for grain yield in soybean breeding.

**Electronic supplementary material:**

The online version of this article (doi:10.1007/s11032-016-0504-9) contains supplementary material, which is available to authorized users.

## Introduction

Soybean [*Glycine max* (L.) Merr.] is one of the most important sources of oil and plant protein (Masuda and Goldsmith 2009). Substantial genetic improvements are required for both traits to feed an estimated world population of 9 billion by 2050 (Ray et al. 2013). Genomic selection (GS) is a novel breeding tool accelerating the selection gain per time unit. GS was initially used for animal breeding (Meuwissen et al. 2001), and its potential is currently intensively studied in plant populations (Heffner et al. 2009; Jannink et al. 2010; Nakaya and Isobe 2012). These experimental studies included data of many major crops such as barley (Zhong et al. 2009), wheat (Rutkoski et al. 2011; Zhao et al. 2015; Pérez-Rodríguez et al. 2012; Crossa et al. 2014), maize (Zhao et al. 2012a, b; Bernardo 2013, 2014), rice (Spindel et al. 2015), sunflower (Reif et al. 2013), forage plants (Hayes et al. 2013), sugar beet (Wurschum et al. 2013), and soybean (Bao et al. 2014; Shu et al. 2013). All studies underline the potential of genomic selection as a powerful tool to accelerate selection gain in plant breeding.

Information on the level of prediction accuracy of genomic selection is crucial to integrate this new tool into applied plant breeding programs. GS prediction accuracy is affected by many factors (Zhong et al. 2009; Calus et al. 2008; Solberg et al. 2008; Zhao et al. 2012a, b; Habier et al. 2007). Thereby, the number of markers is one factor to successfully integrate GS in applied plant breeding programs. A high number of markers facilitate to capture most of the linkage information between QTL and SNP (Solberg et al. 2008; Meuwissen et al. 2001). Nevertheless, large number of markers increases costs and more importantly can create problems due to collinearity among markers. Moreover, as GS also exploits relatedness (Habier et al. 2007, 2010), it is pivotal to have a balanced set of markers allowing to portray reliably the relationship matrix (Liu et al. 2015; Habier et al. 2010).

Soybean is suitable for genomic selection because of moderated genome size and rapid progress on soybean genome sequencing (Schmutz et al. 2010) and re-sequencing (Lam et al. 2010; Li et al. 2013). Moreover, SNP markers have been developed which are distributed throughout the soybean genome (Song et al. 2013) accelerating the application of GS. Shu et al. (2013) used 288 soybean varieties and 79 sequence-characterized amplified region (SCAR) markers and illustrated the potential of whole-genome prediction of hundred-seed weight. Bao et al. (2014) used 282 elite soybean lines, which were fingerprinted with 1536 single nucleotide polymorphism (SNP) markers, and highlighted the prospective of genomic selection for improving resistance to soybean cyst nematode (SCN). All previous research showed that genomic selection was an effective procedure in soybean breeding. However, results on genomic selection in soybean on complex traits such as yield are to the best of our knowledge still missing.

The objectives of this study were to apply ridge regression best linear unbiased prediction in a population of 235 soybean varieties fingerprinted with 5361 genome-wide distributed SNPs in order to (1) explore the genomic prediction accuracy for plant height and yield per plant, (2) discuss the relationship between prediction accuracy and numbers of markers, and (3) evaluate the effect of marker preselection based on different methods on the prediction accuracy.

## Materials and methods

### Field trials

Our study comprised phenotypic data of 235 soybean varieties provided by the National Key Facility for Crop Gene Resources and Genetic Improvement (NFCIR), Institute of Crop Science, Chinese Academy of Agricultural Science. Out of the 235 varieties, 185 were North Spring soybean (NSs) and 50 HuangHuai summer soybean (HHSs) lines. The 235 varieties were evaluated in replicated field trials in 23 locations in Northeast China and in the HuangHuai region in the year 2011 (Supplementary Table S1). The experimental designs were randomized complete block designs with two replications. Plots consisted of three rows with 3 m in length and 0.2 m apart. Fertility and pest management were performed following standard management recommendations. Plant height (cm) and yield per plant (g) were determined in each location following standard protocols (Qiu et al. 2006).

### Phenotypic data analyses

Variance components and heritability of plant height and yield per plant were estimated using the lme4 package implemented in the software package R (Bates et al. 2014). The following mixed linear model was fitted:

$$y_{ij} = \mu + L_{i} + G_{j} + e_{ij} ,$$

where *y*_*ij*_ is the average phenotypic value for *i*th line at *j*th location, *μ* is the population mean, *L*_*i*_ and *G*_*j*_ refer to the effect of *j*th location and *i*th line, respectively, and *e*_*ij*_ denotes the random residual term. Variance components were estimated assuming random location and genotype effects. The best linear unbiased estimation (BLUE) of each line was determined using the same model mentioned above by assuming fixed genotypic effect and random location effects. The difference of target traits average between NSs subsets and HHSs subsets was evaluated applying a *t* test using PASW statistics.

### Genotypic data and linkage disequilibrium analysis

The 235 soybean lines were genotyped with Illumina SoySNP 6 k iSelect BeadChip which comprised 5361 SNPs. These SNPs were chosen from the Illumina SoySNP 50 k iSelect BeadChip (Illumina, San Diego, USA) (Song et al. 2013). We selected SNPs that were located in the proximity of previously described QTLs for various traits. Genotypes are called using the program GenomeStudio (Illumina, San Diego, USA). SNPs with proportion of missing data exceeding 10 % were excluded. For the remaining SNPs, missing values were imputed (Poland et al. 2012). Minor allele frequency (MAF) and polymorphism information content (PIC) were estimated using software PowerMarker version 3.0 (http://www.powermarker.net). Linkage disequilibrium parameter (*r*^2^) between SNP pairs was estimated using the statistical software R (Team 2014) (https://www.r-project.org/). Decay of linkage disequilibrium was explored based on the data of estimated *r*^2^ against genetic distance for all SNP pairs, by fitting a curve with the locally weighted polynomial regression method (Cleveland 1979). To evaluate the population structure, principal component analysis (PCA) was performed using genotypic data. PCA was completed using software TASSEL 3.0 (http://www.maizegenetics.net/). The first two principal components were used to examine the presence of subpopulation structure.

### Genomic selection and cross-validation

The potential of genomic selection was examined focusing on ridge regression best linear unbiased prediction (RR-BLUP) implemented in the statistical package “rrBLUP” (Endelman 2011). Let n be the number of genotypes and p be the number of markers. The RR-BLUP model has the form, where *y* is the vector of BLUEs of genotypic values obtained in the phenotypic data analyses, *µ* refers to the overall mean, *α* is the vector of additive effects of markers, *X* = (*x*_*ij*_) is the *n* *×* *p* matrix of markers with *x*_*ij*_ being the number of a chosen allele at the *j*th locus for the *i*th genotype, and *e* is the vector of residual terms. In the model, we assumed that marker and residual effects are randomly distributed with $\alpha \sim\,N(0,I_{p} \alpha_{\alpha }^{2} )$ and, where *I*_*p*_ and *I*_*n*_ denote identity matrices with respective dimensions, $\alpha_{\alpha }^{2} = {{\alpha_{G}^{2} } \mathord{\left/ {\vphantom {{\alpha_{G}^{2} } p}} \right. \kern-0pt} p}$ and note that $\alpha_{G}^{2}$ and $\alpha_{e}^{2}$ were the estimated genotypic and residual variance components in the phenotypic data analyses, and *l* refers to the number of locations.

We evaluated the prediction accuracy of genomic selection applying fivefold cross-validations. Marker effects were estimated in the training population and the effects were used to predict the genotypic values in the test population. The Pearson product-moment correlation coefficient between the predicted and observed phenotype (*r*_MP_) was estimated, and prediction accuracy (*r*_GS_) was calculated by standardizing *r*_MP_ by the square root of the broad-sense heritability. We repeated the procedure 500 times to reduce the sampling error. In addition, we examined the prediction accuracy also within the North Spring soybean (NSs) subpopulation contrasting it with a random subset of the total population with the same sample size.

### Sampling strategy of markers

#### Random sampling method (RSM)

We randomly sampled SNPs to form different subsets. The number of sampled SNPs varied from 5 to 100 % of the total number of SNPs using five percent intervals. Fivefold cross-validation was applied to study the accuracy of genomic selection with the different subsets. 500 replicates were explored to eliminate sampling error.

#### Haplotype block analysis (HBA)

Haplotype analysis was completed using Haploview 4.2 software based on the population of all 235 soybean lines. Haplotype blocks were defined following previous suggestions (Gabriel et al. 2002). The 5361 SNPs were classified after haplotype block analysis into SNPs belonging to haplotype blocks and SNPs not forming haplotype blocks. We selected then randomly one SNP per haplotype block plus SNPs not forming haplotype blocks. This data were then again used in combination with fivefold cross-validation to study the accuracy of genomic selection. 500 replicates were explored to eliminate sampling error.

#### Evenly sampling method (ESM)

The same numbers of SNPs as used in the haplotype block analyses were selected evenly according to their position around genome. Fivefold cross-validation and 500 replicates were explored to evaluate the prediction accuracy of target traits according to previous scenarios.

## Results

### Extensive phenotyping revealed large genetic variation for plant height and grain yield

We observed for both traits, plant height and grain yield per plant, a significant (*P* < 0.01) and broad genetic variation for the assayed 235 soybean varieties. Lines belonging to the HuangHuai summer group (HHSs) displayed significantly (*P* < 0.01) higher plant height and larger grain yield per plant as compared to North Spring (NSs) lines (Table 1). Heritability estimates of plant height and yield per plant amounted to 0.96 and 0.63, respectively, (Table 1).

**Table 1:** Genetic variance, broad-sense heritability and contrast of plant height (cm) and yield per plant (g) performances between two subpopulations reflecting different ecotypes

Trait	Genetic variance	Heritability	Mean ± SD		*t* value
			NSs^a^	HHSs^b^	
Plant height	253.33**	0.96	60.26 ± 1.1450	92.37 ± 2.4931	−12.66**
Yield per plant	10.80**	0.63	20.94 ± 0.3289	25.42 ± 0.5174	−6.71**

** Significantly different at 0.01 level probability

^a^North Spring soybean

^b^HuangHuai Summer soybean

### Analysis of linkage disequilibrium identified haplotype blocks comprising up to 22 SNPs

**Fig. 1:**
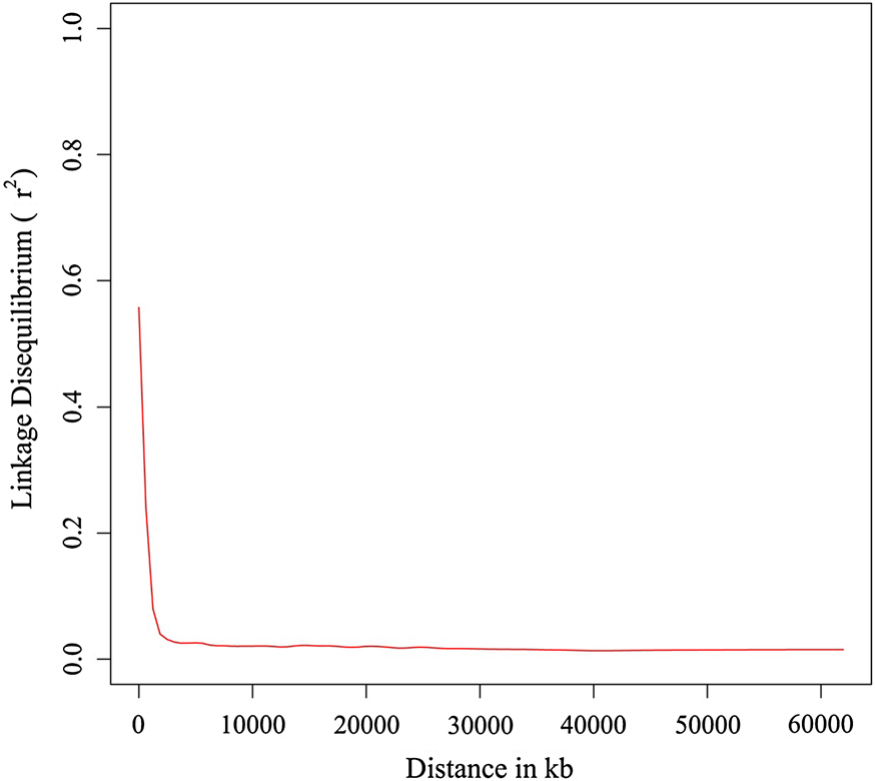
Decay of linkage disequilibrium (*r* ^2^) with physical map distances between markers. The curve was fitted using locally weighted polynomial regression

**Fig. 2:**
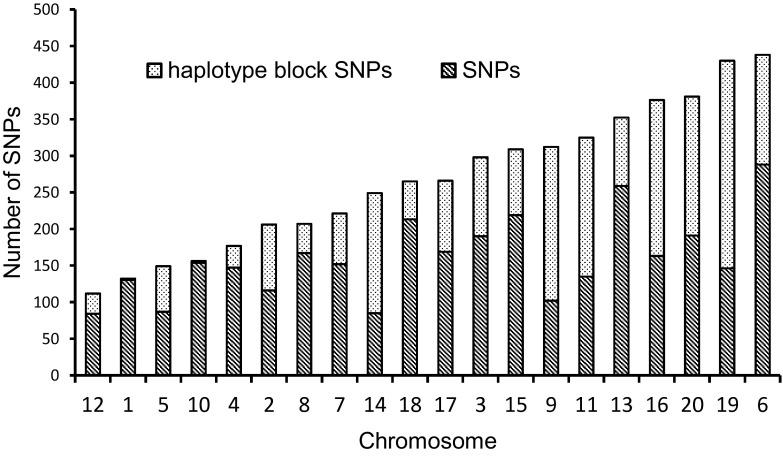
Distributions of haplotype block SNPs and SNPs for the 20 soybean chromosomes

Linkage disequilibrium between pairs of SNPs declined sharply to *r*^2^ = 0.1 at around 1000 kb (Fig. 1). We identify 357 haplotype blocks across the 20 soybean chromosomes, which comprised a total of 2164 SNPs. The remaining 3197 SNPs, which were not forming haplotype blocks, were defined as “SNPs”. The number of SNPs composing haplotype blocks ranged from 2 to 22 and the percentage of SNPs assigned to haplotype blocks in every chromosome ranged from 1.28 % (chromosome 1) to 67.31 % (chromosome 9), respectively, (Fig. 2).

### Population structure analysis revealed presence of genetically distinct subpopulations

**Fig. 3:**
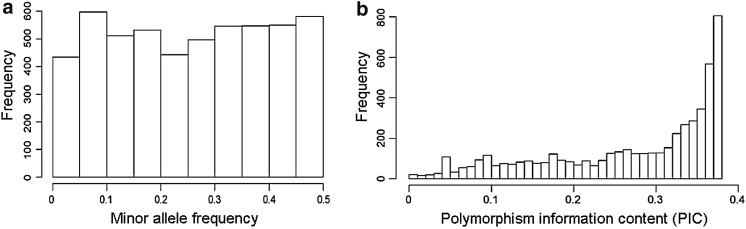
**a** Histogram of minor allele frequency and **b** polymorphism information content of 5275 SNPs

**Fig. 4:**
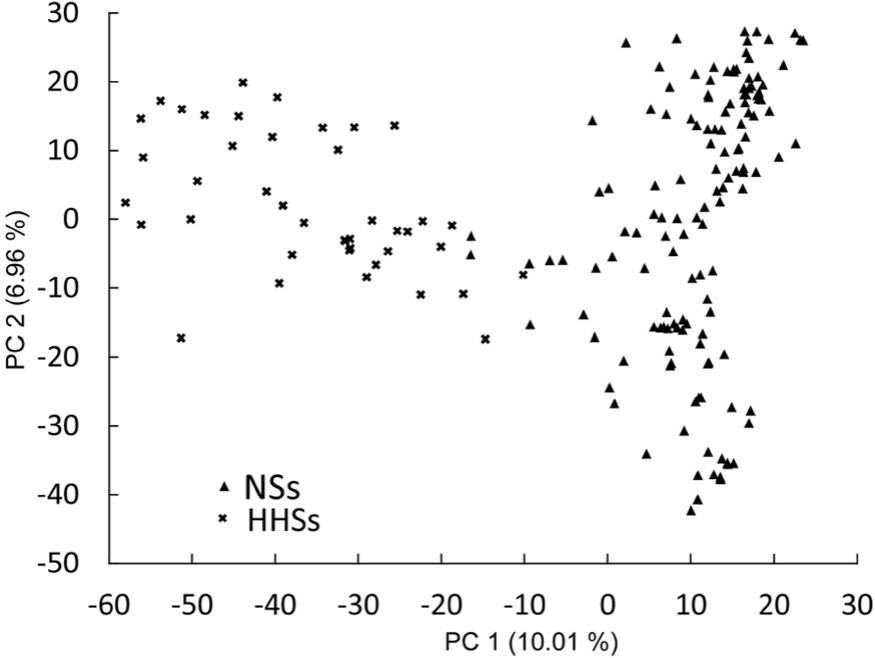
Scatter plots of the first two principal components (PC) for 235 soybean varieties clustered into North Spring soybean (NSs) and Huanghuai Summer soybean (HHSs) subpopulations

After quality filtering, 5275 SNPs were used to explore the population structure of the 235 soybean varieties. The minor allele frequency averaged 0.25 (Fig. 3a) and PIC values averaged 0.27 (Fig. 3b). The first two principle components explained in total 17 % of the molecular variation. The scatter plot using the first two principle components revealed presence of two genetically distinct subpopulations (Fig. 4). Soybean varieties of different ecotypes were separated into two subsets according to the first principle component.

### Genomic prediction accuracies were high for plant height and moderate for grain yield

**Fig. 5:**
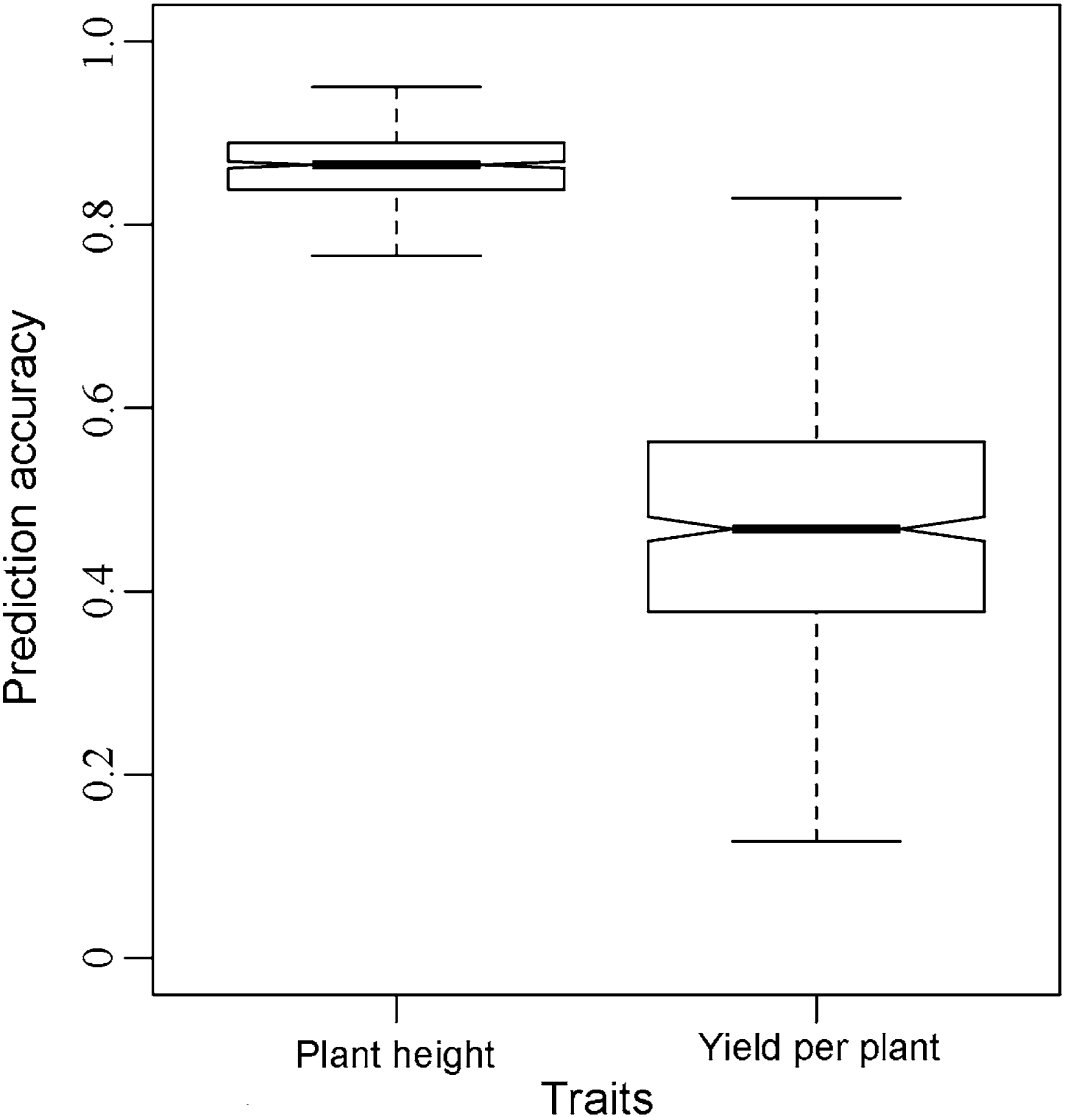
Box-Whisker plots of cross-validated prediction accuracies of plant height and yield per plant, with the method of ridge regression best linear unbiased prediction

We used fivefold cross-validation to examine the potential of genome-wide prediction for different soybean traits. The average prediction accuracy was substantially higher for plant height (*r*_GS_ = 0.86) compared to yield per plant (*r*_GS_ = 0.47) (Fig. 5, Table S2). Moreover, the standard deviation of the prediction accuracies was substantially larger for yield per plant compared to plant height (Fig. 5).

### Preselection of markers slightly enhanced genomic prediction accuracy for grain yield

**Fig. 6:**
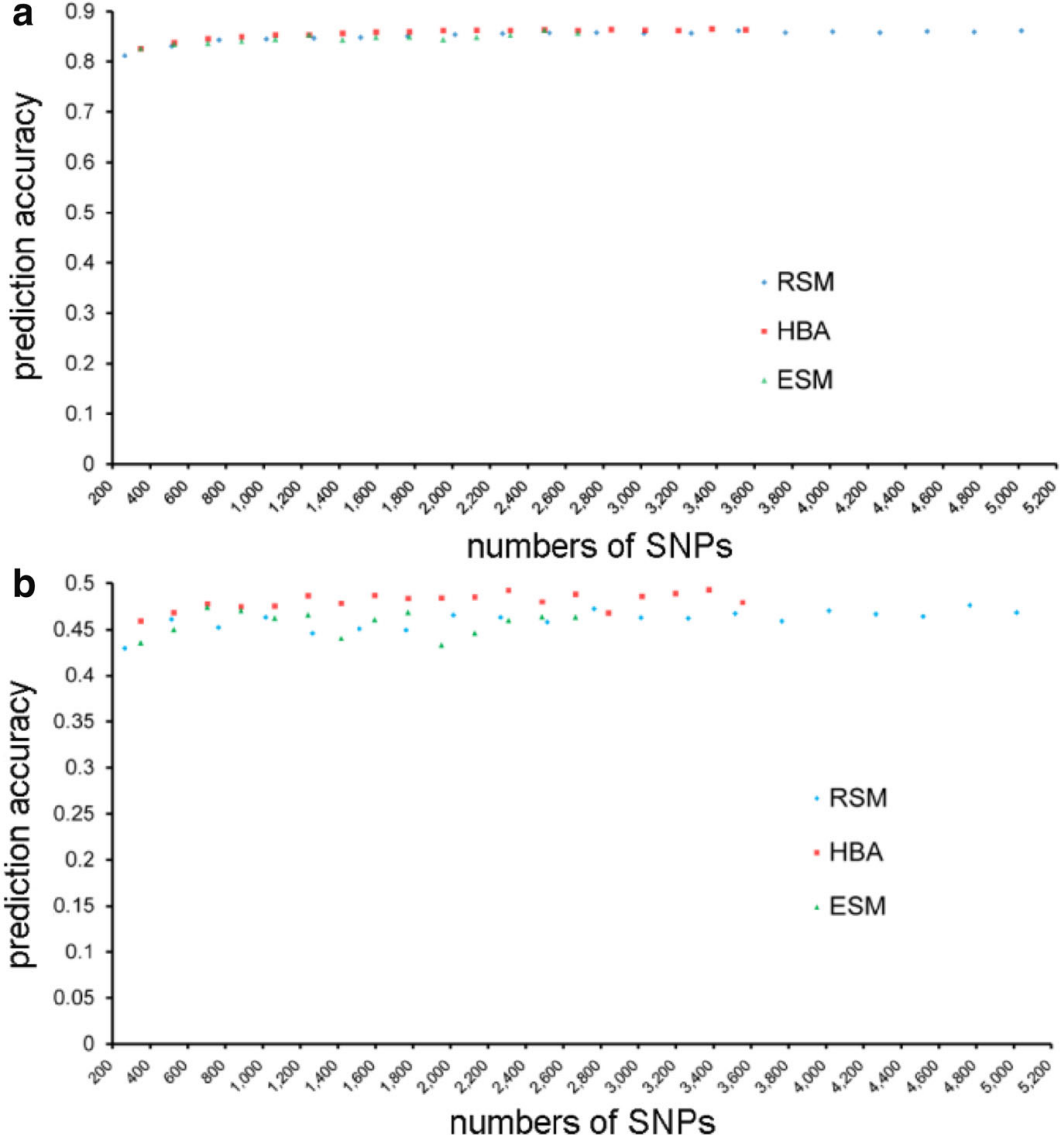
Cross-validated prediction accuracies of ridge regression best linear unbiased prediction based on three marker sampling strategies for plant height (**a**) and yield per plant (**b**). Marker subsets were selected using a random sampling (RSM), a haplotype block-based sampling strategy (HBA), and evenly sampling method (ESM)

We studied the effects of different marker sampling strategies on genomic prediction accuracy for a broad range of marker densities. The marker sampling strategies were a random sampling method (RSM), a haplotype block analysis-based sampling (HBA), and evenly sampling method (ESM). Using a step of 250 SNPs, 265 to 5015 SNPs were randomly selected for RSM in order to estimate the prediction accuracies (Supplementary Table S2). In contrast, for HBA we selected one SNP for each of the 357 identified haplotype blocks. These SNPs were combined with the remaining 3197 “SNPs”. From this data set, we randomly selected 172 to 3554 SNPs with a step of 178 SNPs and examined the prediction accuracy for the target traits (Supplementary Table S2). We also selected from 172 to 2664 SNPs evenly around genome with a step of 178 SNPs for ESM strategy and evaluated the prediction accuracies (Supplementary Table S2). Generally, prediction accuracies for both plant height and yield per plant increased with increasing number of SNPs for both sampling strategies (Fig. 6, Supplementary Table S2). Haplotype block analysis-based sampling facilitated highest prediction accuracies for both target traits. Randomly sampling method improved the prediction accuracy slightly compared with ESM. For yield per plant, prediction accuracy based on markers selected with HBA increased by 3.66 and 4.10 % compared with the RSM and ESM strategies, respectively. In contrast, for plant height, prediction accuracies were comparable for all marker selection strategies.

## Discussion

### Population structure impaired the prediction accuracy depending on the target trait

Pronounced population structure has to be considered when evaluating the potential of genomic selection (Hayes et al. 2009; Guo et al. 2014; Isidro et al. 2015). In our study, a total of 235 soybean varieties were sampled reflecting two distinct ecotypes (Fig. 4). Consequently, prediction accuracies within the subpopulations of the two distinct ecotypes are potentially overestimated using cross-validations based on the total population. To study this in more detail, we also estimated the prediction accuracies within the larger subpopulation North Spring (NSs) comprising 185 lines. We found that prediction accuracies decreased by 5.27 and 67.07 % for plant height and yield per plant, respectively, using the North Spring soybean subset compared to the total population using a standardized training population size. Consequently, the population structure substantially influenced the prediction accuracy for yield per plant and has to be considered when interpreting the results. If the wish is to develop soybean varieties for breeding programs specifically designed for the North Spring target environments, the prediction accuracies for yield per plant are upward biased. In contrast, plant height is not affected by subpopulation structure, and thus results of the total population are also applicable for breeding programs specifically targeting North Spring environments.

### Genomic selection is a promising tool for soybean breeding

As important agronomic traits, the prediction accuracies of plant height and yield were explored in maize (Zhao et al. 2012a; Riedelsheimer et al. 2012; Crossa et al. 2013), wheat (Heffner et al. 2011; Poland et al. 2012), rye (Wang et al. 2014), barley (Sallam et al. 2015), and rice (Spindel et al. 2015). The previously reported prediction accuracies ranged from 0.34 to 0.85 for plant height and from 0.17 to 0.87 for yield. Our results with prediction accuracies of 0.87 for plant height and 0.49 for yield per plant (Fig. 5) are lying within the range of these previously reported values. The higher prediction accuracies for plant height as compared to yield can be explained by a less complex genetic architecture of plant height than yield (Heffner et al. 2011; Spindel et al. 2015; Sallam et al. 2015).

Different strategies completely or partially relying on genomic selection have been proposed to be implemented into breeding programs (Longin et al. 2015; Bassi et al. 2016). The choice of the most suited strategy thereby depends on the prediction accuracy achieved by the genomic selection models. At early selection stages, many individuals are commonly evaluated at a limited number of locations focusing on negative selection, i.e., disregarding the inferior genotypes (He et al. 2016). Genomic selection is for this early selection stages an interesting alternative if costs of genotyping are comparable to the costs of a single location yield trial (Heffner et al. 2010). We observed for grain yield a prediction accuracy of 0.47 in our study corresponding to field trials conducted at 3–4 locations (Supplementary Table S2, Fig. 5). Consequently, genomic selection is for yield per plant an interesting alternative for negative selection, thus, replacing early stages of selection in soybean breeding. This trend of favoring genomic selection for negative selection of grain yield has been also observed for other crops such as wheat (He et al. 2016).

Breeding programs exclusively based on genomic predictions focusing also on positive selection, i.e., identifying the best genotype, were only recommended if high prediction accuracies can be achieved by the genomic selection models (Longin et al. 2015). The observed prediction accuracy for plant height amounted to 0.86 in our study (Supplementary Table S2, Fig. 5). Thus, plant height can be reliably predicted based on genomic selection alone.

### Effects of marker sampling strategy on genomic prediction accuracies

Meuwissen (Meuwissen 2009) showed in a simulation study that to take advantages of high marker densities, comprehensive training data sets exhibiting a large effective population size are required. Elite soybean breeding populations, however, display often a limited effective population size (St Martin 1982). In this case, marker density may be reduced with only marginal loss in prediction accuracies for an economic implementation of genomic selection. We compared in our study different strategies to reduce the marker density. Our findings show that the marker sampling strategy impacted the prediction accuracies only marginally for plant height (Fig. 6a). In contrast, for grain yield, prediction accuracies based on markers selected with HBA increased by approximately 4 % compared with the two alternative strategies examined in our study (Fig. 6b). Thus, applying marker preselection based on haplotype blocks is an interesting option for a cost-efficient implementation of genomic selection for grain yield in soybean breeding.

## Electronic supplementary material

Below is the link to the electronic supplementary material.

Supplementary material 1 (DOCX 23 kb)

## Abbreviations

GS: Genomic selection
SNP: Single nucleotide polymorphism
rrBLUP: Ridge regression best linear unbiased prediction
RSM: Random sampling method
HBA: Haplotype block analysis
ESM: Evenly sampling method
