# Supplementary material for: Potential of marker selection to increase prediction accuracy of genomic selection in soybean (Glycine max L.)
Source: Mol Breed. 2016 Jul 28;36:113. doi: 10.1007/s11032-016-0504-9 (PMC4965486; doi:10.1007/s11032-016-0504-9)
Supplement: Supplementary file 1 — Supplementary material 1 (DOCX 23 kb) [file 11032_2016_504_MOESM1_ESM.docx]

**Supplementary Information**

S1 Geographical information of field trials locations

| Ecoregion | Location | Latitude | Longitude |
| --- | --- | --- | --- |
| North Spring soybean | Jiusan | 48.88 | 125.25 |
|  | Moqi | 48.47 | 124.50 |
|  | Zhalantun | 48.01 | 122.74 |
|  | Yargenchu | 47.75 | 122.62 |
|  | Jiamusi | 46.80 | 130.32 |
|  | Suihua | 46.64 | 126.98 |
|  | Harbin | 45.80 | 126.53 |
|  | Dehui | 44.52 | 125.68 |
|  | Shulan | 44.41 | 126.97 |
|  | Jiaohe | 43.72 | 127.34 |
|  | Tonghua | 41.49 | 125.92 |
| HuangHuai Summer soybean | Beijing | 39.90 | 116.41 |
|  | Shijiazhuang | 38.04 | 114.51 |
|  | Dezhou | 37.43 | 116.36 |
|  | Weifang | 36.71 | 119.16 |
|  | Jinan | 36.67 | 116.99 |
|  | Handan | 36.63 | 114.54 |
|  | Xuchang | 34.04 | 113.85 |
|  | Zhoukou | 33.63 | 114.64 |
|  | Mengcheng | 33.27 | 116.56 |
|  | Longkang | 33.13 | 116.90 |
|  | Fu'nan | 32.66 | 115.60 |
|  | Zhengyang | 32.61 | 114.39 |

S2 Relationships between number of SNPs and prediction accuracies (r_GS_) using different marker sampling strategies

| Trait | Sampling strategy | | | | | |
| --- | --- | --- | --- | --- | --- | --- |
|  | Randomly sampling method (RSM) | | Haplotype block analysis (HBA) | | Evenly sampling method (ESM) | |
|  | SNP amount | r_GS_ ± SE | SNP amount | r_GS_ ± SE | SNP amount | r_GS_ ± SE |
| Plant height | 265 | 0.8123 ± 0.0024 | 172 | 0.8030 ± 0.0024 | 172 | 0.7986 ± 0.0024 |
|  | 515 | 0.8308 ±0.0021 | 350 | 0.8261 ± 0.0022 | 350 | 0.8265 ±0.0021 |
|  | 765 | 0.8425 ± 0.0020 | 528 | 0.8380 ± 0.0019 | 528 | 0.8347 ± 0.0020 |
|  | 1015 | 0.8445 ± 0.0019 | 706 | 0.8453 ± 0.0021 | 706 | 0.8366 ± 0.0021 |
|  | 1265 | 0.8468 ± 0.0019 | 884 | 0.8492 ± 0.0018 | 884 | 0.8409 ± 0.0019 |
|  | 1515 | 0.8477 ± 0.0019 | 1062 | 0.8530 ± 0.0019 | 1062 | 0.8449 ± 0.0019 |
|  | 1765 | 0.8503 ± 0.0019 | 1240 | 0.8531 ± 0.0019 | 1240 | 0.8527 ± 0.0017 |
|  | 2015 | 0.8537 ± 0.0018 | 1418 | 0.8562 ± 0.0018 | 1418 | 0.8437 ± 0.0018 |
|  | 2265 | 0.8552 ± 0.0018 | 1596 | 0.8584 ± 0.0016 | 1596 | 0.8483 ± 0.0018 |
|  | 2515 | 0.8565 ± 0.0018 | 1774 | 0.8590 ± 0.0018 | 1774 | 0.8494 ± 0.0018 |
|  | 2765 | 0.8571 ± 0.0017 | 1952 | 0.8618 ± 0.0017 | 1952 | 0.8441 ± 0.0020 |
|  | 3015 | 0.8561 ± 0.0019 | 2130 | 0.8622 ± 0.0017 | 2130 | 0.8487 ± 0.0018 |
|  | 3265 | 0.8562 ± 0.0018 | 2308 | 0.8616 ± 0.0017 | 2308 | 0.8531 ± 0.0018 |
|  | 3515 | 0.8607 ± 0.0017 | 2486 | 0.8627 ± 0.0017 | 2486 | 0.8625 ± 0.0017 |
|  | 3765 | 0.8571 ± 0.0018 | 2664 | 0.8619 ± 0.0017 | 2664 | 0.8565 ± 0.0018 |
|  | 4015 | 0.8590 ± 0.0017 | 2842 | 0.8637 ± 0.0017 |  |  |
|  | 4265 | 0.8571 ± 0.0017 | 3020 | 0.8624 ± 0.0017 |  |  |
|  | 4515 | 0.8595 ± 0.0017 | 3198 | 0.8616 ± 0.0017 |  |  |
|  | 4765 | 0.8583 ± 0.0018 | 3376 | 0.8650 ± 0.0017 |  |  |
|  | 5015 | 0.8609 ± 0.0017 | 3554 | 0.8630 ± 0.0018 |  |  |
| Yield per plant | 265 | 0.4292 ± 0.0064 | 172 | 0.4438 ± 0.0063 | 172 | 0.4256 ± 0.0067 |
|  | 515 | 0.4607 ± 0.0064 | 350 | 0.4591 ± 0.0064 | 350 | 0.4355 ± 0.0066 |
|  | 765 | 0.4520 ± 0.0058 | 528 | 0.4679 ± 0.0063 | 528 | 0.4499 ± 0.0061 |
|  | 1015 | 0.4628 ± 0.0062 | 706 | 0.4776 ± 0.0062 | 706 | 0.4738 ± 0.0058 |
|  | 1265 | 0.4460 ± 0.0060 | 884 | 0.4744 ± 0.0063 | 884 | 0.4705 ± 0.0061 |
|  | 1515 | 0.4505 ± 0.0063 | 1062 | 0.4752 ± 0.0062 | 1062 | 0.4623 ± 0.0061 |
|  | 1765 | 0.4492 ± 0.0062 | 1240 | 0.4866 ± 0.0063 | 1240 | 0.4657 ± 0.0059 |
|  | 2015 | 0.4654 ± 0.0059 | 1418 | 0.4782 ± 0.0064 | 1418 | 0.4405 ± 0.0061 |
|  | 2265 | 0.4628 ± 0.0062 | 1596 | 0.4869 ± 0.0063 | 1596 | 0.4607 ± 0.0062 |
|  | 2515 | 0.4578 ± 0.0063 | 1774 | 0.4837 ± 0.0060 | 1774 | 0.4684 ± 0.0060 |
|  | 2765 | 0.4722 ± 0.0061 | 1952 | 0.4843 ± 0.0059 | 1952 | 0.4327 ± 0.0063 |
|  | 3015 | 0.4627 ± 0.0063 | 2130 | 0.4854 ± 0.0061 | 2130 | 0.4462 ± 0.0066 |
|  | 3265 | 0.4619 ± 0.0061 | 2308 | 0.4926 ± 0.0060 | 2308 | 0.4598 ± 0.0061 |
|  | 3515 | 0.4669 ± 0.0059 | 2486 | 0.4799 ± 0.0063 | 2486 | 0.4637 ± 0.0062 |
|  | 3765 | 0.4589 ± 0.0061 | 2664 | 0.4885 ± 0.0063 | 2664 | 0.4633 ± 0.0064 |
|  | 4015 | 0.4702 ± 0.0061 | 2842 | 0.4676 ± 0.0061 |  |  |
|  | 4265 | 0.4664 ± 0.0061 | 3020 | 0.4860 ± 0.0064 |  |  |
|  | 4515 | 0.4639 ± 0.0062 | 3198 | 0.4890 ± 0.0062 |  |  |
|  | 4765 | 0.4758 ± 0.0061 | 3376 | 0.4932 ± 0.0058 |  |  |
|  | 5015 | 0.4681 ± 0.0060 | 3554 | 0.4794 ± 0.0061 |  |  |
